# Is anemia an independent risk factor for postpartum depression in women who have a cesarean section? - A prospective observational study

**DOI:** 10.1186/s12884-018-2032-6

**Published:** 2018-10-11

**Authors:** Nirmala Chandrasekaran, Leanne R De Souza, Marcelo L Urquia, Beverley Young, Anne Mcleod, Rory Windrim, Howard Berger

**Affiliations:** 1 Department of Maternal and Fetal Medicine, St Michaels Hospital, 30 Bond street, Toronto, ON M5B 1W8 Canada; 2 Li Ka Shing Knowledge Institute, St Michael’s Hospital, Toronto, Canada; 3 Perinatal Mental Health Program, Mount Sinai Hospital, Toronto, Canada; 4 Sunnybrook Health Sciences, Toronto, Canada; 5 Mt Sinai Hospital, Toronto, Canada

**Keywords:** Anemia, Iron stores, Cesarean section, Postpartum depression, Functional status

## Abstract

**Background:**

The symptoms of anemia and depression are very similar suggesting that there may be an association between the two entities. The aim of this study is to assess whether postpartum anemia (PPA) is an independent risk factor for de novo postpartum depression (PPD)in women undergoing elective cesarean section.

**Methods:**

Women after an uncomplicated term cesarean section were recruited and their hemoglobin and iron status were measured on day 3–5 post section and again at 6 weeks. Postpartum depression was screened using the Edinburgh Postnatal Depression Scale (EPDS) and functional capacity was assessed with the RAND 12-item Health survey.

**Results:**

One hundred and three women completed the study. The incidence of probable postpartum depression (PPD) as defined by EPDS score ≥ 10 was 17% at 6 weeks. There was no difference in hemoglobin or iron status in women who had PPD compared to those without (OR-0.69; 95% CI-0.15-2.49). Similarly, there was no significant association between low hemoglobin and maternal functional status (OR -1.03; 95% CI-0.34 - 2.94).

**Conclusions:**

Neither anemia or low iron stores were found to be an independent risk factors for postpartum depression or decreased postpartum functional capacity in women who undergo an elective cesarean section.

## Background

Postpartum anemia (PPA) is a serious health condition affecting approximately 27% of North American women during the early postpartum period (puerperium). Among women with PPA, an estimated 50% are also iron deficient, wherein iron stores are depleted while hemoglobin could be normal or low [1–3]. Maternal consequences of anemia are well known and include cardiovascular symptoms like palpitations and dizziness, fatigue, reduced physical and mental performance, reduced immune function, reduced peripartum blood reserves and increased risk of blood transfusion [4].

Postpartum depression (PPD) is one of the most common conditions in the postnatal period affecting between 10 and 15% of women [5]. The causes of postpartum depression remain unclear and risk factors include a family or personal history of depression before pregnancy, low income, poor social support, poor interfamilial relationships, low self-esteem, parental stress, unwanted or unplanned pregnancy and recent stressful life events in a woman’s life [6, 7]. Up to 50% of women with PPD will go on to develop PPD in subsequent pregnancies, and their offspring are at increased risk of impaired psychological and intellectual development [8, 9]. The classic symptoms associated with PPD are depressed mood, anxiety, anhedonia, appetite and sleep disturbances, physical agitation, fatigue, feelings of worthlessness and excessive guilt, decreased concentration and recurrent thoughts of death or suicidal ideation [10].

Symptoms of PPD are common to those found in women with PPA. Whether a causal pathway relates PPD and PPA, is unknown, however identifying a physiological link between these two conditions may help identify women at increased risk of PPD. Such an association has been suggested in the literature but there are few studies that specifically address the association between postpartum anemia and postpartum depression [11–13]. Furthermore the association between PPA and maternal functional status, defined by an individual’s ability to perform normal daily activities required to meet basic needs, fulfill usual roles, and maintain health and well-being has not been previously studied in the context of developing PPD.

Moreover, some studies show that women who undergo cesarean delivery could potentially be at increased risk of developing PPD possibly due to perceived pain and immobility of recovery, stress response to surgery and perceived failure to undergo vaginal birth as potential reasons for the association. A meta-analysis of the effects of cesarean delivery on the risk of PPD concluded that although the risk of PPD was higher with both elective and emergency Cesarean sections compared to vaginal delivery, no statistically significant association was identified for elective section and PPD [14].

We aimed to determine whether anemia and iron deficiency, or the combination of these, are independent risk factors for newly diagnosed postpartum depression and decreased functional capacity in women who deliver by elective cesarean section.

## Methods

The study was approved by the Research and Ethics board (REB) of St Michaels Hospital (REB #08–155). Informed written consent was obtained from all the women that agreed to participate in the study.

All women 18 years of age or older, with a full term, singleton pregnancy (37–41 weeks’ gestation), undergoing elective Caesarean section were eligible to enroll in the study. Emergency Caesarean sections were excluded to avoid potential obstetric confounders associated with adverse labor and delivery experiences. Women were excluded from the study if they had symptomatic anemia necessitating blood transfusion, significant fetal anomalies, preexisting severe chronic maternal illness (e.g. cardiac disease with functional class >II, chronic renal failure), preexisting maternal depression or other psychiatric illness, preexisting hemoglobinopathy (e.g. thalassemia, sickle cell disease) or confounding social factors (e.g. single parent or no fixed address).

The primary outcome for our study was the incidence of probable postpartum depression determined by the Edinburgh postpartum depression scale (EPDS). The secondary outcomes were the association of PPD with measures of iron stores, measured by hemoglobin and soluble transferrin receptor and functional status measured by the RAND test.

Eligible women were approached within 24 h of delivery and followed prospectively for 6 weeks. Baseline demographic (age, ethnicity, gestational age) and clinical data (use of prenatal vitamins or iron supplements and any medical comorbidities) were collected, followed by venous blood collection for hemoglobin (gm/dl), ferritin (ng/ml) and soluble transferrin receptor (mg/L) levels. The intention to breastfeed was documented. Anemia was defined as hemoglobin < 110 g/l as described by the World Health Organization (WHO, 2007) [15]. A venous blood draw was repeated at 3 weeks postpartum to assess hemoglobin and ferritin levels. Women identified with anemia were prescribed iron supplements, with compliance monitored using a pill counter. At 6 weeks postpartum, a thyroid function test was administered to exclude postpartum thyroiditis as a potential confounder.

Functional status was assessed by administering the RAND 36-item health survey (SF-36). The RAND is a validated questionnaire that evaluates 8 health concept sub scales that include physical functioning, bodily pain, role limitations due to physical health problems, role limitations due to personal or emotional problems, emotional well-being, social functioning, energy/fatigue, and general health perceptions that may affect quality of life [16, 17]. From these 8 individual subscales, 2 overall scores were generated: physical health component (PCS) and mental health component (MCS). The scoring for the 8 domains and the 2 overall components are based on a 0–100 General Health Rating index scale, with a higher value corresponding to a higher quality of life [16].

The EPDS was administered at enrollment, 24 h after elective Cesarean section and again at 3 and 6 weeks postpartum. A score of ≥10 on the EPDS was considered positive for postpartum depression, as this has previously been shown to be highly sensitive for the detection of minor depression. Despite its intended use as a screening tool, the EPDS is widely accepted as a validated method to detect postpartum depression and the cut off threshold score of 10, readily identifies all cases of major depression with a negative predictive value of 99.7% [18–21]. Patients with a score ≥ 10 in the EPDS or > 2 in question 10 (thoughts of self-harm), were referred to a staff psychiatrist for the administration of the gold standard diagnostic tool - the Structured Clinical Interview for DSM-III-R (SCID, nonpoint version) and treatment if deemed necessary.

A lactation consultant contacted each woman at 2, 4 and 6 weeks postpartum to inquire about the frequency of breastfeeding or bottle-feeding and supplementation with formula among women who were breastfeeding. Women who reported rare supplementation with formula were coded as predominantly breastfeeding. Among those who reported exclusive bottle feeding, the number of weeks of any breastfeeding was recorded.

### Statistical analysis

Previous studies indicate approximately 13% of women are diagnosed with major or minor PPD [5]. Using a 13% incidence of PPD among non-anemic patients, a sample size of 100 women would detect a 2.8-fold increase in PPD (37%) or higher in the defined anemic group (hemoglobin < 110), with a statistical power of 80% and alpha 0.05. This magnitude of increased risk is similar to the risk observed for other accepted risk factors for PPD [e.g. History of psychiatric disease OR 3.1 (2.3–4.2; Social isolation OR 3.6 (1.9–7.0)] [22] and would thus be a clinically significant effect size. This sample size also offers 80% power to detect a 50% increase in mean EPDS score.

Fisher’s exact test was used to test the association between categorical variables and PPD and a T-test assessed continuous variables. We also used exact logistic regression to obtain odds ratios with 95% confidence intervals. Linear regression was used for the comparison of means between groups.

Given the lower than expected incidence of anemia in the study cohort, data were stratified according to the lowest quartiles to enable a comprehensive description of the effects of hemoglobin and iron status on functional capacity and postpartum depression. In multivariable analyses, we controlled for maternal age at delivery, parity, prenatal intake of vitamins and continuation of breastfeeding, each collected at 6 weeks postpartum. Data was analyzed with SAS 9.4 (SAS Institute Inc., Cary, NC).

## Results

**Fig. 1:**
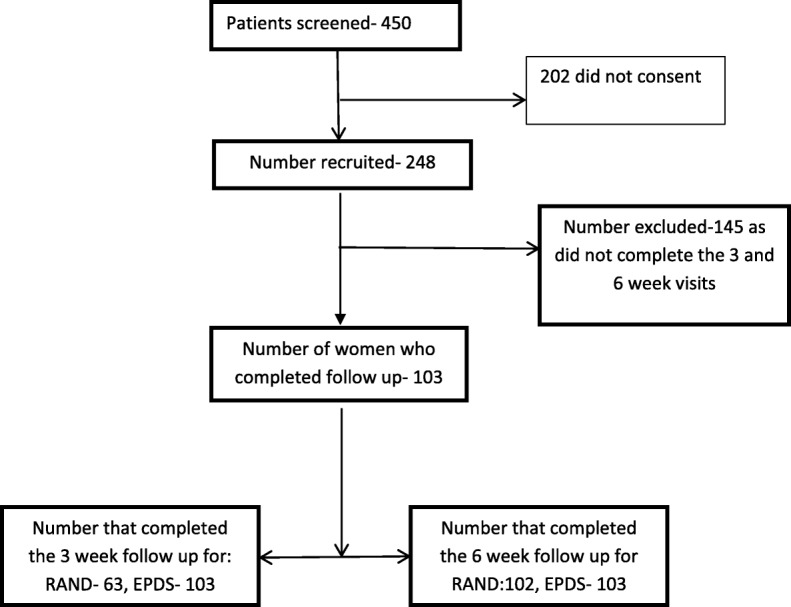
Flow chart of the recruitment process

A total of 248 women were recruited for the study, with 145 lost to follow-up (Fig. 1). The excluded women were not demographically different from the study group (data not shown). Demographic characteristics of the 103 women are as listed in Table 1. There were no statistical differences in the baseline characteristics of women who screened positive for PPD and those who did not have any indication of PPD (Table 1).

**Table 1:** Characteristics of the study population

Maternal characteristics	EPDS ≥10 at any visit (PPD = yes)	EPDS < 10 at any visit (PPD = no)	*p*-value**
N (%)	18(17.5%)	85 (82.5%)	
Age: mean (SD)	34.1 (4.1)	34.9 (4.3)	0.46
Nulliparous (%)	2/18 (11%)	31/85 (36%)	0.17
Number of previous livebirths or stillbirths: mean (SD)	0.7 (0.8)	0.9 (0.8)	0.36
Gestation weeks at delivery: mean (SD)	38.9 (0.6)	38.8 (0.9)	0.72
Less than 13 years of education, N (%)	3 (16.7)	4 (4.7)	0.11
Ethnicity (non-White) *N = 102	10 (55.6)	36 (42.9)	0.44
Pregnancy complications (GDM, PIH, IUGR and Preeclampsia)	6 (33.3)	23 (27.1)	0.58
Smoking during pregnancy *N = 102	0 (0.00)	1 (1.19)	1.00
alcohol use during pregnancy *N = 102	5 (27.8)	9 (10.7)	0.07
Illegal Drug use during pregnancy*N = 102	0 (0.00)	1 (1.19)	1.00
Birth weight: N, mean (SD)	3334 (401)	3370 (470)	0.76
BMI: N; mean (SD)	25.9 (6.9)	25.6 (5.5)	0.84
Prenatal Vitamins (%)	12 (66.7)	60 (70.6)	0.78
Breastfeeding (%)	15 (83.3)	76 (89.4)	0.44

*Indicates total N, excluding missing/unknown values for row characteristics (1 subject in the PPD = yes group)

**Fisher’s test for proportions, 2-sided

The incidence of probable postpartum depression in our study population was 17% at 6 weeks postpartum. There were 14 women (13%) who were anemic prior to the Cesarean section. There were no cases of anemia identified at the 6-week follow-up visit. Patients who scored > 10 on EPDS and > 2 on question 10 (self-harm) were referred to a psychiatrist. Not all patients accepted the referral. The 3 patients who accepted the referral had the diagnosis confirmed and were commenced on SSRIs.

### Anemia indices and PPD

The incidence of postpartum depression was not different in anemic vs. non-anemic mothers (Table 2).

**Table 2:** Associations between anemia indicators, functional status and postpartum depression

Maternal characteristics	N	EPDS > = 10 at any visit (PPD = yes *N* = 18)	EPDS < 10 at any visit (PPD = no *N* = 85)	Odds Ratio^a^ (q1 vs. rest) or mean differences (95% CI)	Adjusted Odds Ratio^b^ (q1 vs. rest) or mean differences (95% CI)
Anemia indicators					
HGB quartile 1 at pre-c/section), N (%)	103	3 (16.67)	27 (31.76)	0.43^a^ (0.07 to 1.72)	0.44^b^ (0.08 to 1.74)
HGB quartile 1 at visit 1 N (%)	101	3 (16.67)	24 (28.92)	0. 50^a^ (0.08 to 1.98)	0.53^b^ (0.09 to 2.12)
Mean HGB at pre-c/section (SD)	103	124.0 (9.9)	119.2 (9.2)	4.8 (−0.4 to 10.0)	4.6 (−0.2 to 9.5)
Mean HGB at visit 1 (SD)	101	109.4 (9.6)	105.2 (9.7)	4.2 (−0.4 to 10.0)	3.8 (−1.3 to 8.8)
HGB quartile 1 at visit 3, N (%)	103	4 (22.22)	25 (29.41)	0.69^a^ (0.15 to 2.49)	0.73^b^ (0.15 to 2.82)
Mean HGB at visit 3 (SD)	103	133.4 (8.1)	129.0 (8.1)	4.4 (0.2 to 8.5)*	4.2 (0.1 to 8.2)*
Delta Hgb_visit 1 - Hgb_presection (note 1)	101	− 13.9 (9.0)	−14.0 (8.3)	−0.1 (− 6.7 to 6.5)	− 1.1 (− 6.9 to 4.7)
Delta Hgb_visit 3 - Hgb_presection (note 2)	103	8.1 (9.8)	10.0 (8.9)	1.9 (−3.6 to 7.4)	−1.0 (− 7.2 to 5.3)
Delta Hgb_visit 3-Hgb visit 1 (note 2)	101	22.7 (11.09)	24.1 (9.2)	1.4 (−4.4 to 7.2)	− 0.2 (− 6.8 to 6.3)
Ferritin quartile 1 at visit 1, N (%)	103	6 (33.33)	20 (23.53)	1.62^a^ (0.44 to 5.42)	1.80^b^ (0.46 to 6.60)
Mean ferritin at visit 1 (SD)	103	48.8 (27.7)	46.7 (25.4)	2.12 (−11.2 to 15.4)	0.9 (− 12.7 to 14.4)
Ferritin quartile 1 at visit 3, N (%)	103	3 (16.67)	23 (27.06)	0.54^a^ (0.09 to 2.18)	0.52^b^ (0.09 to 2.20)
Mean ferritin at visit 3 (SD)	103	54.9 (41.0)	42.7 (29.6)	12.3 (−4.1 to 28.7)	13.1 (−3.5 to 29.7)
Soluble transferrin receptor quartile 1 at visit 1, n (%)	103	3 (16.67)	24 (28.24)	0.51^a^ (0.09 to 2.05)	0.46^b^ (0.08 to 1.87)
Mean Soluble transferrin receptor at visit 1 (SD)	103	1.21 (0.33)	1.14 (0.43)	0.1 (−0.1 to 0.3)	0.1 (− 0.1 to 0.3)
Rand, quartile 1 at visit 3, n (%)	102	8 (44.44)	19 (22.35)	2.75^a^ (0.82 to 9.04)	3.24^b^ (0.92 to 11.70)
Exclusive Breastfeeding at visit 3, n (%)	103	10 (55.56)	59 (69.41)	0.55 (0.17 to 1.82)	0.39*
Rand mean score at visit 3 (SD)	102	83.7449 15.1247	89.1667 13.1908	−5.4 (−12.4 to 1.6)	−5.8 (−12.7 to 1.1)

*P-value < 0.05; ^a^Unadjusted Odds ratio; ^b^Odds ratio adjusted for age, parity, prenatal vitamins, breastfeeding at week 6

There was no statistical difference in hemoglobin, ferritin and soluble transferrin receptor levels between women who had a positive screen for PPD and those who did not have PPD according to EPDS scores.

To assess a possible effect of acute blood loss on PPD, we calculated the change in pre-operative hemoglobin values to those at 6 weeks’ post-partum (ΔHb). There was no significant association between ∆Hb and the presence of PPD (Table 2). There were also no significant differences in the thyroid stimulating hormone levels between the women with and without a positive screen for post-partum depression.

Functional capacity as assessed by the RAND score did not significantly differ between the two groups (Table 2) and, scores on the RAND showed no relation to the scores on the EPDS.

### Anemia indices and postpartum functional capacity

There is no established cut off threshold values for RAND scores. Therefore, we compared the lowest RAND score quartile to the upper three quartiles. As indicated in Table 3, there was no correlation between either the hemoglobin or the iron store indices with measures of functional status.

**Table 3:** Associations between anemia indicators and postpartum functional status

Maternal characteristics	N	RAND Quartile 1 at visit 3 (*n* = 27)	RAND Quartiles 2, 3 and 4 at visit 3 (*N* = 76)	Odds Ratio^a^ (q1 vs. rest) or mean differences (95% CI)	Adjusted Odds Ratio^b^ (q1 vs. rest) or mean differences (95% CI)
Anemia indicators					
HGB quartile 1 at pre-csection, N (%)	103	8 (29.63)	22 (28.95)	1.03^a^ (0.34 to 2.94)	1.13^b^ (0.37 to 3.28)
Mean HGB (pre-csection) (SD)	103	120.3 (11.16)	119.9 (8.84)	0.4 (−4.6 to 3.9)	0.1 (−4.2 to 4.4)
HGB quartile 1 at visit 1, N (%)	101	7 (25.93)	20 (27.03)	0.95^a^ (0.29 to 2.80)	1.01^b^ (0.30 to 3.12)
Mean HGB at visit 1 (SD)	101	105.9 (8.88)	105.9 (10.16)	0.01 (−4.4 to 4.4)	0.04 (−4.6 to 4.5)
HGB quartile 1 at visit 3, N (%)	103	10 (37.04)	19 (25.00)	1.75^a^ (0.61 to 4.92)	2.44^b^ (0.76 to 8.00)
Mean HGB at visit 3 (SD)	103	129.2 (9.20)	130.0 (7.89)	−0.8 (−2.9 to 4.5)	−1.8 (−5.4 to 1.7)
Delta Hgb_visit 1 - Hgb_presection (note 1)	101	−14.4 (7.9)	−13.9 (8.6)	−0.5 (−4.3 to 3.2)	− 0.3 (− 4.2 to 3.5)
Delta Hgb_visit 3 - Hgb_presection (note 2)	103	8.9 (11.2)	10.1 (8.1)	−1.2 (−5.2 to 2.8)	− 1.9 (−6.0 to 2.2)
Delta Hgb_visit 3-Hgb visit 1 (note 2)	101	23.3 (7.9)	24.2 (10.0)	−0.9 (−5.2 to 3.3)	−1.9 (− 6.2 to 2.4)
Ferritin quartile 1 at visit 1, N (%)	103	5 (18.52)	21 (27.63)	0.60^a^ (0.16 to 1.91)	0.77^b^ (0.19 to 2.70)
Mean ferritin at visit 1 (SD)	103	50.4 (26.1)	45.9 (25.7)	4.5 (−6.9 to 16.0)	5.0 (−6.9 to 16.8)
Ferritin quartile 1 at visit 3, N (%)	103	5 (18.52)	21 (27.63)	0.60^a^ (0.16 to 1.91)	0.71^b^ (0.18 to 2.39)
Mean ferritin at visit 3 (SD)	103	50.5 (35.2)	42.8 (30.8)	7.7 (−6.5 to 21.9)	5.5 (−9.2 to 20.2)
Soluble transferrin receptor quartile 1 at visit 1, n (%)	103	8 (29.63)	19 (25.00)	1.26^a^ (0.41 to 3.65)	1.26^b^ (0.41 to 3.65)
Mean Soluble transferrin receptor at visit 1 (SD)	103	1.1 (0.3)	1.2 (0.5)	−0.1 (−0.3 to 0.1)	−0.1 (−0.2 to 0.1)

^a^Unadjusted Odds ratio; ^b^Odds ratio adjusted for age, parity, prenatal vitamins, breastfeeding at week 6

Exact binary logistic regression analysis showed that neither anemia nor iron status independent predictor of postpartum depression or post-partum functional status (Table 2).

## Discussion

We found that neither anemia nor iron stores measured postpartum are associated with postpartum depression or functional status postpartum.

The biological association between PPD and PPA could be attributed to iron deficiency rather than decreased hemoglobin levels. Iron is critical for adequate myelination, neurotransmitter metabolism and function, and neuronal cellular and oxidative processes and through these processes, may contribute to the development of clinical depression [23]. The association between anemia and poor functional status is well established in the non-pregnant population and has been attributed to poor muscle oxygenation and reduced exercise tolerance. To date, there have been no studies that evaluated this association in the pregnant population.

While iron deficiency and anemia have been related to poor cognition, depression and reduced functional capacity in the non-pregnant population, there have been conflicting results in studies examining pregnant populations. In one study [11], the authors used the Center for Epidemiological Studies-Depressive Symptomatology Scale (CES-D) to screen for symptoms of depression on day 28 postpartum and reported that hemoglobin concentration on day 7 to be negatively correlated with self-reported depressive symptoms on day 28, however this study had a relatively small sample size and did not measure iron status or thyroid function. In contrast, other research reported no correlation between low hemoglobin levels and high Edinburgh test for post-partum depression (EPDS) scale [12]. Possible cohort effects may impede extrapolation of study findings as those with low hemoglobin were also more likely to have had an abnormal delivery and a consequent propensity for PPD regardless of degree of anemia. A randomized control trial demonstrated an improvement in emotional and cognitive measures at 9 months postpartum in iron treated anemic women but not in placebo controls [13]. A study examining risk factors for postpartum psychosis demonstrated anemia in 51.4% of cases with postpartum psychosis. However, there is no information about the incidence of anemia in a control group of women without postpartum psychosis. These results are most likely not applicable to the postpartum population in developed countries and cannot be extrapolated to patients with PPD.

Another study illustrates no relation between maternal iron stores and postpartum depression [24]. The incidence of postpartum depression in this population was significantly higher (> 20%) and again the authors did not control for hemoglobin. Some authors have suggested that anemia, but not iron deficiency is associated with a higher incidence of postnatal depression [25]. But this study was limited by a small sample size. Also, the incidence of PPD was significantly lower (5.5%) compared to what is quoted in the literature. A similar finding was reported in a recent study from Saudi Arabia by Alharbi et al. [26].They found that anemia but not iron deficiency is a significant risk factor for postpartum depression, but this study had a higher than normal incidence of PPD (33%) and the investigators did not control for confounders like traumatic delivery or history of preexisting depression.

In a recent nested cohort study, the authors demonstrate a positive association between anemia at discharge from the maternity ward and the development of PPD symptoms, even after controlling for plausible confounders such as previous psychological contact, experience of delivery, mood during pregnancy and no exclusive breastfeeding at 6 weeks postpartum (OR = 2.29, 95%CI = 1.15–4.58) [27].

Anemia has been shown to be a possible risk factor for reduced functional status and is well studied in cardiac, elderly and cancer patients but there are no studies addressing this issue in pregnant patients.

The strengths of this study include the use of a prospective study design, limiting the cohort to women who had an elective Caesarean section which eliminated potential obstetric confounders that have known associations with postpartum depression. The exclusion of women with preexisting depression was a strength as this is a known risk factor and permitted the determination of de novo postpartum depression in a cohort of low risk women. We also controlled for the continuation of breast feeding as there is evidence that there is reduced risk of PPD in women who continue to breast feed [28]. In addition, we controlled for thyroid function - a well-known confounder for postpartum depression. The use of soluble transferrin receptor levels allowed us to more accurately define true iron deficiency. This is first study to our knowledge that explored the effects of anemia and iron status on functional scores in Obstetric population.

Our limitations include a potential selection bias as only 103 out of the 248 women recruited completed the planned follow up. The incidence of anemia and iron deficiency in our population was lower than expected, thereby limiting the variability in the exposure and an ability to detect significant effects, if they exist. We attempted to overcome this by analyzing hemoglobin using the lowest quartile values. There is the possibility of an association between maternal anemia (or iron deficiency) and the risk of developing PPD, but this association will manifest only at certain threshold values. Given that all anemic women post Cesarean section were treated with iron supplements, there were no anemic patients at the 6 weeks visit when the second EPDS was administered. The results reported herein are applicable only to women undergoing elective Cesarean sections and it is possible that there would be different results in women having CS in labour or vaginal delivery which need to be explored further. Finally, there is also a possibility that the women who did not complete the 3 and 6 weeks follow up might have been affected by postpartum depression that the study did not capture.

## Conclusion

We did not find any evidence of an association between postpartum anemia and postpartum depression, despite this association occurring in the non-pregnant population. Further research is needed to ascertain why iron status relates to these symptoms in some contexts but not in others.

## Abbreviations

EPDS: Edinburgh Postnatal Depression Scale
PPA: Postpartum anemia
PPD: Postpartum depression
